# Ecotoxicological Effects of the Anionic Surfactant Sodium Dodecyl Sulfate (SDS) in Two Marine Primary Producers: *Phaeodactylum tricornutum* and *Ulva lactuca*

**DOI:** 10.3390/toxics10120780

**Published:** 2022-12-13

**Authors:** Ricardo Cruz de Carvalho, Eduardo Feijão, Ana Rita Matos, Maria Teresa Cabrita, Andrei B. Utkin, Sara C. Novais, Marco F. L. Lemos, Isabel Caçador, João Carlos Marques, Patrick Reis-Santos, Vanessa F. Fonseca, Bernardo Duarte

**Affiliations:** 1 MARE–Marine and Environmental Sciences Centre, ARNET–Aquatic Research Network Associate Laboratory, Faculdade de Ciências da Universidade de Lisboa, Campo Grande, 1749-016 Lisbon, Portugal; 2 cE3c–Centre for Ecology, Evolution and Environmental Changes, Faculty of Sciences, University of Lisbon, Campo Grande, Edifício C2, Piso 5, 1749-016 Lisbon, Portugal; 3 Departamento de Biologia Vegetal, Faculdade de Ciências da Universidade de Lisboa, Campo Grande, 1749-016 Lisboa, Portugal; 4 BioISI–Instituto de Biosistemas e Ciências Integrativas, Faculdade de Ciências, Universidade de Lisboa, 1749-016 Lisboa, Portugal; 5 Centro de Estudos Geográficos (CEG), Instituto de Geografia e Ordenamento do Território (IGOT), Universidade de Lisboa, Rua Branca Edmée Marques, 1600-276 Lisboa, Portugal; 6 Laboratório Associado TERRA, Edifício Prof. Azevedo Gomes, Instituto Superior de Agronomia, Tapada da Ajuda, 1349-017 Lisboa, Portugal; 7 INOV-INESC, Rua Alves Redol 9, 1000-029 Lisboa, Portugal; 8 CeFEMA, Universidade de Lisboa, Av. Rovisco Pais 1, 1049-001 Lisboa, Portugal; 9 MARE–Marine and Environmental Sciences Centre, ARNET–Aquatic Research Network Associate Laboratory, ESTM, Polytechnic of Leiria, 2520-641 Peniche, Portugal; 10 MARE–Marine and Environmental Sciences Centre, ARNET–Aquatic Research Network, Department of Life Sciences, University of Coimbra, 3000 Coimbra, Portugal; 11 Southern Seas Ecology Laboratories, School of Biological Sciences, The University of Adelaide, Adelaide, SA 5005, Australia; 12 Departamento de Biologia Animal, Faculdade de Ciências, Universidade de Lisboa, Campo Grande, 1749-016 Lisboa, Portugal

**Keywords:** anionic surfactant detergents, biomarkers, ecotoxicity, marine phototrophs, photobiology

## Abstract

Sodium Dodecyl Sulfate (SDS) is an anionic surfactant, extensively used in detergents, household and personal care products, as well as in industrial processes. The present study aimed to disclose the potential toxicological effects of SDS exposure under environmentally relevant concentrations (0, 0.1, 1, 3, and 10 mg L^−1^) on the physiology and biochemistry (photosynthesis, pigment, and lipid composition, antioxidative systems, and energy balance) of two marine autotrophs: the diatom *Phaeodactylum tricornutum* and the macroalgae *Ulva lactuca*. A growth rate (GR) reduction in *P. tricornutum* was observed with a classic dose-response effect towards the highest applied concentration, while a GR increase occurred in *U. lactuca*. Regarding photochemistry, the decrease in the fluorescence of the OJIP curves and laser-induced fluorescence allowed a better separation between SDS treatments in *U. lactuca* compared with *P. tricornutum*. Although all pigments significantly decreased in *U. lactuca* at the highest concentrations (except for antheraxanthin), no significant variations occurred in *P. tricornutum*. On the other hand, changes in fatty acid content were observed in *P. tricornutum* but not in *U. lactuca*. In terms of classical biomarker assessment, a dose-effect relationship of individual biomarkers versus SDS dose applied; *U. lactuca* displayed a higher number of biomarker candidates, including those in distinct metabolic pathways, increasing its usefulness for ecotoxicological applications. By evaluating the potential application of optical and biochemical traits, it was evident that the fatty acid profiles of the different exposure groups are excellent candidates in *P. tricornutum*, concomitant with the characteristics of this anionic surfactant. On the other hand, the results presented by laser-induced fluorescence and some parameters of PAM fluorometry in *U. lactuca* may be an advantage in the field, offering non-invasive, fast, easy-to-use, high-throughput screening techniques as excellent tools for ecotoxicology assessment.

## 1. Introduction

The oceans support the livelihood of many communities. However, nowadays they face several threats, such as unsustainable exploitation and anthropogenic pollution [1,2]. New contaminants continue to emerge with technological development. These belong to diverse classes of natural or artificial compounds, which are not usually included in monitoring routines [3]. Emergent pollutants have diverse and miscellaneous uses, ranging from cosmetics, pesticides, and pharmaceutical products, to cleaning and disinfection and many others, leading to their daily discharge into the environment, and representing a contemporary challenge due to unknown environmental and human health impacts [4,5]. The study of these anthropogenic inputs is crucial to gather information to mitigate the lack of guidelines regarding emerging pollutants.

Surfactants are extensively used every day as household products or part of industrial processes. They are characterized by having an amphiphilic structure, which translates into two different functional groups within the same molecule, one polar (e.g., sulfate (–OSO_3_^−^), glyceryl group) and another nonpolar (e.g., alkyl chain with 8–22 carbons). These compounds can be divided into two classes, ionic and non-ionic, due to the ionization capacity of the hydrophilic group in the first case [6].

Anionic surfactants are the most common and abundant components in a cleansing formulation. By the mid-2000s, 12.5 million tons of surfactants were produced worldwide, and in the last decade, in Europe, 1.22 Mt of anionic surfactants were used annually and profusely discharged into the environment [7,8,9]. Detergents, at low concentrations, alter membrane permeability by denaturation and binding proteins leading to cell permeabilization and lysis; at higher concentrations, they lead to progressive loss of cell bioactivity since they can remove all the phospholipids from the cell membrane [10,11,12,13]. Because cells are composed of membrane systems, the impact of anionic surfactants with micellar activity becomes an environmental concern, due to their ability to destroy the lipid lamellar structure [14]. One of the most commonly used anionic surfactants is Sodium Dodecyl Sulfate (SDS), being one of the simplest carbon chain structures. Chemically, SDS is an organosulfate (CH_3_(CH_2_)_11_SO_4_Na), described as an alkyl group with a terminal sulphate group that enables the formation of micelles and acts as a detergent [6,15]. Although SDS has been classified as “eco-friendly” due to its readily biodegradable properties [16,17], it has been persistently found in water bodies and soils, due to the high consumption of consumer products (e.g., laundry products, shampoo, toothpastes), where it is very difficult to remove due to its adsorption properties [9,18,19].

Microalgae, such as *Phaeodactylum tricornutum*, are responsible for about 20% of global primary photosynthetic production, oxygen production, and carbon uptake, sustaining a whole ecosystem [20,21,22] and providing various essential metabolites such as long-chain polyunsaturated fatty acids (LC-PUFAs), namely omega-3 and -6 fatty acids to higher trophic levels [23]. In addition, previous work indicates that anthropogenic waste impairs marine diatom photosynthetic metabolism; any disturbance in the ecosystem can be readily detected by monitoring these organisms in a rapid, sensitive, and cost-effective way [24,25,26,27,28].

Macroalgae are greatly represented amongst the primary producers, being a critical component of the trophic web and forming part of the diet of many marine animal species [29] with high carbohydrate and protein content, and a low lipid content [30]. The macroalgae *Ulva lactuca* (Ulvaceae, Chlorophyta), commonly known as sea lettuce, is ubiquitous in coastal benthic communities around the world, in marine and estuarine environments [31,32], having a highly charged cell wall composed of the mucopolysaccharide ulvan, which allows the adsorption of pollutants [33]. Since this species responds directly to environmental pollution, it has been used in ecotoxicology bioassays (e.g., UlvaTox) for metals (e.g., silver, arsenic, cadmium, cobalt, chromium, copper, iron, mercury, manganese, nickel, lead, and zinc); disinfectants (e.g., formalin); fossil fuels (e.g., diesel); medicines (e.g., thiazolidinediones); fungicides (e.g., tributyltin oxide); and herbicides (e.g., glyphosate) [34,35,36,37,38,39,40].

The presence of anionic surfactants can have daily, monthly and seasonal variations in wastewater treatment plants, reaching concentrations between 1.15 mg L^−1^ and 10.6 mg L^−1^ [11,41]. More recently, with the COVID-19 pandemic and the ability of these products to deactivate SARS-CoV-2 [42,43], the production and occurrence of SDS in the environment are expected to increase significantly [44]. In this context, the present study aims to assess the ecotoxicological effects of SDS exposure in two marine phototrophs, the diatom *P. tricornutum* and the macroalgae *U. lactuca* as model organisms, exposed to environmentally relevant concentrations. To address the physiological effects of this anionic surfactant in these key primary producers, but also aiming to disclose which species might be the best candidate for potential biomonitoring, a wide array of biochemical (pigment and fatty acid profiles, oxidative stress, energetic metabolism) and biophysical (photochemistry) assays were performed.

## 2. Materials and Methods

### 2.1. Experimental Setup

Monoclonal cultures of the diatom *Phaeodactylum tricornutum* Bohlin (Bacillariophyceae) (IO 108–01, IPMA) were grown for 4 days under controlled conditions (14 h light:10 h dark photoperiod, at 18 ± 1 °C, under constant aeration) in 250 mL of F/2 medium in a growth chamber (Fytoscope FS130, PSI, Dásov, Czech Republic) programmed with a sinusoidal function simulating sunrise and sunset (RGB 1:1:1, Maximum PAR 80 μmol photons m^−2^ s^−1^, 14/10 h day/night rhythm) [45]. Initial cell density was 2.7 × 10^5^ cells mL^−1^, according to the algae bioassay’s guidelines [46]. Cultures were allowed to enter the exponential phase for 48 h after which they were exposed to the abovementioned SDS concentrations for an additional 48 h [47,48]. The chosen SDS concentrations (0, 0.1, 1, 3 and 10 mg L^−1^) mimicked environmentally relevant concentrations [11,41,49,50,51]. At the end of the exposure period, samples were collected for analysis, by centrifugation (4000× *g* for 15 min at 4 °C), and after supernatant disposal, instantly frozen using liquid nitrogen and stored at −80 °C until further analysis. All concentrations and tests were performed in triplicate.

The macroalgae *Ulva lactuca* was acquired from AlgaPlus (Ílhavo, Portugal). Before the assay, the macroalgae were kept for one week in an aquarium with a culture medium (filtered seawater enhanced with Provasoli’s Enriched Medium, salinity 36 PSU) [52] under the same controlled conditions, except for maximum PAR (40 μmol photons m^−2^ s^−1^). Disks with a 1.5 cm diameter were cut and, for the assay, introduced into transparent plates with twenty-four wells of the same diameter. The disks were exposed to the abovementioned SDS concentrations. For each SDS concentration group, three replicates were made. For each biochemical analysis, three replicates of each concentration group were used.

### 2.2. Growth Rates and Inhibition

For *P. tricornutum*, cell counting was performed daily using an improved Neubauer counting chamber under 400× times magnification in an inverted microscope (Olympus B×50, Tokyo, Japan). To determine the mean specific growth rate per day, the difference between the initial and final logarithmic cell densities, divided by the exposure period, was determined [53]. For *U. lactuca*, the growth rate was determined by gently drying the disks with paper towel and weighing the biomass of the macroalgae disks at the end of the assay (FW_f_) and a comparison made with the initial weight (FW_i_):

(1) $$\mathrm{GR}=\frac{{\ln\;\mathrm{FW}}_{f}-\ln{\mathrm{FW}}_{i}}{\mathrm{time}\;\mathrm{of}\;\mathrm{exposure}}$$

For each analysis, three replicates were collected from a total of fifteen experimental units [46].

### 2.3. Chlorophyll a Pulse Amplitude Modulated (PAM) Fluorometry

Photobiological assessments were performed through chlorophyll fluorescence analysis (PAM fluorometry) with a FluoroPen FP100 (Photo System Instruments, Drásov, Czech Republic), on a 1 mL cuvette (for *P. tricornutum*) or with leaf clips (for *U. lactuca*), in dark-adapted samples. The OJIP test was used to determine the chlorophyll transient light curves [40,47,48,54], from which the photochemical parameters (Table 1) were obtained according to this method and the equations provided in [55,56,57].

### 2.4. Laser-Induced Fluorescence (LIF) Analysis

In *U. lactuca* disks, the fluorescence emission spectra were measured according to [58] using an LIF sensor built around a frequency-doubled Nd:YAG laser (Quantel, model Ultra 532 30 20HN) and a custom low-noise photodetector described previously [59,60].

### 2.5. Pigment Analysis

Pigment extraction was carried out in sample pellets from *P. tricornutum*, and freeze-dried (48 h) *U. lactuca* disks, according to previous works [40,47]. Pigment analysis was performed according to [61], allowing the detection of chlorophyll *a* (Chl *a*) and *c* (Chl *c*), pheophytin *a* (Pheo *a*), β-carotene (b-carot), fucoxanthin (Fx), diadinoxanthin (DD), and diatoxanthin (DT) in *P. tricornutum*, and chlorophyll *a* (Chl *a*) and *b* (Chl *b*), pheophytin *a* (Pheo *a*), lutein, β-carotene (b-carot), zeaxanthin (Zea), antheraxanthin (Anthera), violaxanthin (Viola), and auroxanthin (Auro) in *U. lactuca*.

### 2.6. Fatty Acid Profiles

For the fatty acid (FA) analysis, the protocol described by Feijão et al. [48] was followed. For the determination of FA unsaturation levels, the double bond index (DBI) was used:

(2) $$\mathrm{DBI}=\frac{2\times \left(\%\mathrm{monoenes}\;+2\times \%\mathrm{dienes}+3\times \%\mathrm{trienes}+4\times \%\mathrm{tetraenes}+5\times \%\mathrm{pentaenes}\right)}{100}$$

### 2.7. Oxidative Stress

Antioxidant enzyme extraction was performed as described in previous works [40,47]. Protein content was determined according to the Bradford method [62]. The activities of catalase (CAT, EC 1.11.1.6), ascorbate peroxidase (APX, EC 1.11.1.11), and superoxide dismutase (SOD, EC 1.15.1.1) were quantified through spectrophotometric methods, as previously described [63,64,65]. The oxidative stress ratio was calculated as follows:

(3) $$\mathrm{Oxidative}\;\mathrm{stress}\;\mathrm{ratio}=\frac{\mathrm{SOD}\;\mathrm{Activity}}{\mathrm{APX}\;\mathrm{Activity}+\mathrm{CAT}\;\mathrm{Activity}}$$

Lipid peroxidation was determined according to [66] with proper amendments [26,67]. Absorbance was registered at 532 and 600 nm and TBARS concentrations were determined through the molar extinction coefficient, 155 mM^−1^ cm^−1^ [66] and expressed as mM of malondialdehyde (MDA) cell^−1^ (*P. tricornutum*), or mM MDA g^−1^ FW (*U. lactuca*).

### 2.8. Energy Balance

To each *P. tricornutum* cell pellet, 1 mL of milli-Q water was added for homogenization by ultrasonication. For *U. lactuca*, the homogenization was made using a mortar and pestle. The lipid, protein and carbohydrate contents were determined by spectrophotometry (Biotek^®^ Instrument, Winooski, VT, USA) following the protocol of [68] with minor modifications in line with [69,70]. Available energy (Ea) was assessed by converting the total protein, carbohydrate and lipid contents using their respective energy combustion into their energetic equivalents [71]. Regarding energy consumption (Ec), the electron transport system (ETS) activity was measured by spectrophotometric methods according to King and Packard [72] with modifications [68]. Cellular energy allocation (CEA) was determined as [73]:

(4) CEA = Ea/Ec

where:

Ea (available energy) = carbohydrate + lipid + protein (mJ 10^−6^ cells, or mJ mg^−1^ FW)

Ec (energy consumption) = ETS activity (mJ h^−1^ 10^−6^ cells, or mJ h^−1^ mg^−1^ FW)

### 2.9. Statistical Analysis

To assess Spearman’s correlation coefficients and significance, the corrplot package was used [74] and barplots were plotted using the ggplot2 package [75]. Due to a lack of normality and homogeneity of variances in the obtained data, non-parametric Kruskal–Wallis with Bonferroni posthoc tests were performed using the agricolae package [76] to assess the differences among the SDS exposure levels for each evaluated variable. Canonical analysis of principal coordinates (CAP) was used to evaluate the ability to successfully classify individuals according to the SDS exposure concentrations using each of the considered biochemical and biophysical traits (Primer 6 software) [77]. All other statistical analyses were performed in R-Studio 1.4.1717.

## 3. Results

### 3.1. Growth Rate

**Figure 1:**
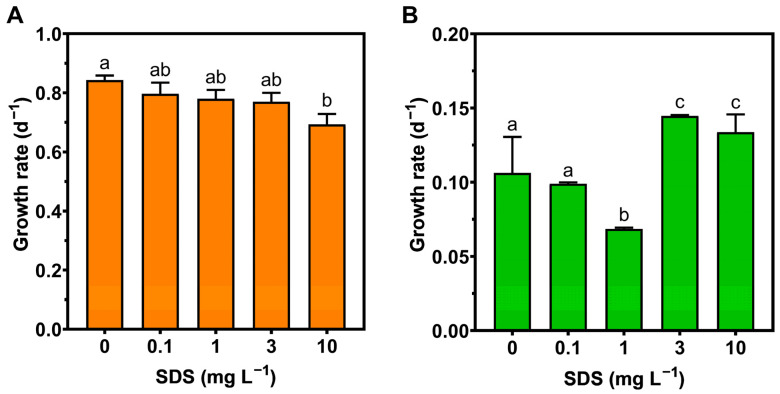
Growth rate of *Phaeodactylum tricornutum* (**A**) and *Ulva lactuca* (**B**) following 48-h exposure to increasing SDS concentrations (mean ± s.d., *n* = 3 per treatment, different letters denote significant differences between treatments at *p* < 0.05).

In *Phaeodactylum tricornutum*, the growth rate (GR) registered the lowest values at the highest SDS concentration (10 mg L^−1^) (Figure 1A). On the other hand, *U. lactuca* also presented a growth rate response to SDS concentration, decreasing at 1 mg L^−1^, but increasing at the highest concentrations (3 and 10 mg L^−1^) (Figure 1B). Overall, growth parameters in *P. tricornutum* showed stronger correlations with exogenous SDS exposure, than in *U. lactuca*.

### 3.2. Photochemistry

**Figure 2:**
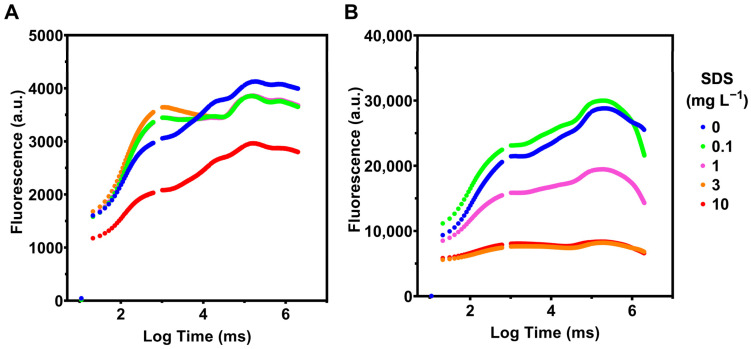
Chlorophyll transient kinetics (OJIP curves or Kautsky plots) in (**A**) *Phaeodactylum tricornutum* and (**B**) *Ulva lactuca* following 48-h exposure to increasing SDS concentrations (mean, *n* = 3).

The chlorophyll transient kinetics (OJIP curves or Kautsky plots) in *P. tricornutum* decreased only at the highest SDS concentration (10 mg L^−1^) (Figure 2A). On the other hand, in *U. lactuca*, the fluorescence emission decreased with increasing exposure to increasing concentrations of SDS, except for 0.1 mg L^−1^, which presents a slight increase relative to the control (Figure 2B). In *U. lactuca* there is a clear separation between exposure to low, intermediate, and high concentrations of SDS.

**Figure 3:**
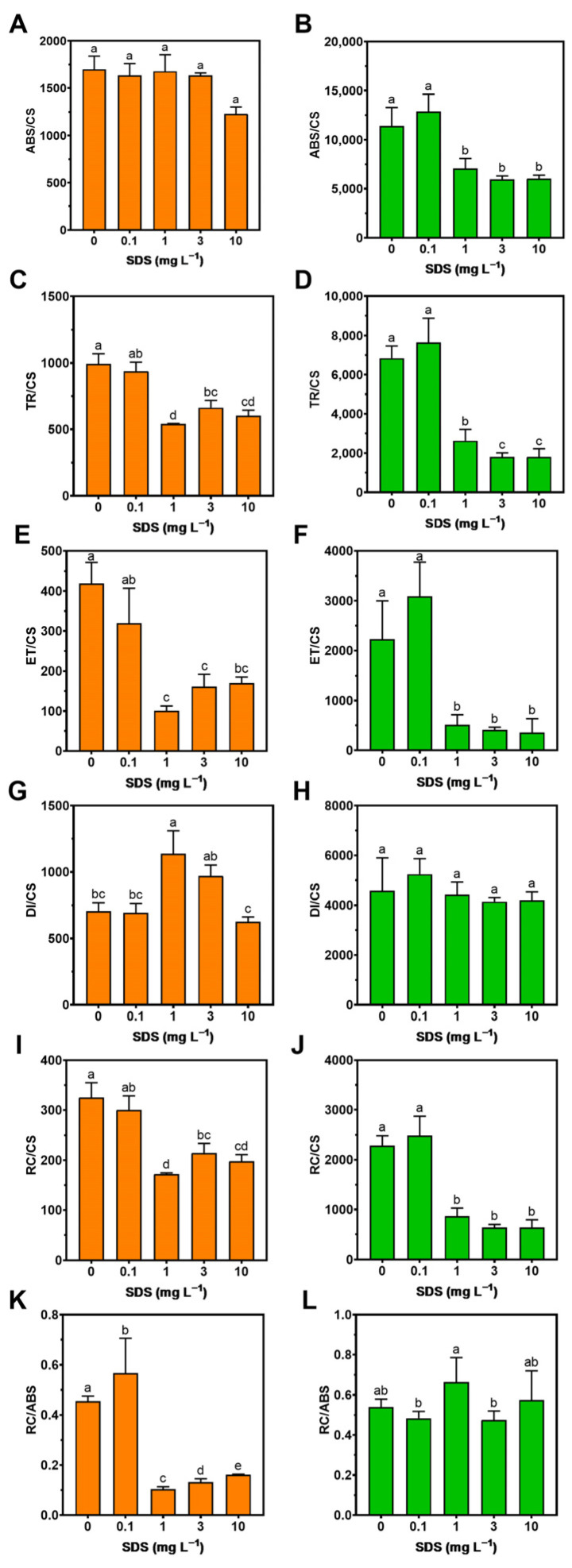
Energy transduction energy fluxes: absorbed (ABS/CS), trapped (TR/CS), transported (ET/CS) and dissipated (DI/CS), the number of oxidized PS II reaction centres per cross-section (RC/CS), and the reaction centre II density within the antenna chlorophyll bed of PS II (RC/ABS) in *Phaeodactylum tricornutum* (**A**,**C**,**E**,**G**,**I**,**K**) and *Ulva lactuca* (**B**,**D**,**F**,**H**,**J**,**L**) following a 48-h exposure to SDS rising concentrations (mean ± s.d., *n* = 3 per treatment, different letters denote significant differences between treatments at *p* < 0.05).

To characterize the overall photochemical process, the previous curves were processed, allowing us to assess changes in the main representative fluxes concerning light-harvesting electronic transport with SDS exposure (Figure 3). Between the control and the SDS exposure concentration, both phototrophic species start to show similar decreases in some of those parameters, starting at 1 mg L^−1^ of SDS, although at different sites of the electron transport chain. Therefore, both species present four parameters that respond to increasing SDS concentrations: TR/CS, ET/CS, RC/CS, and RC/ABS in *P. tricornutum* (Figure 3C,E,I,K, respectively); and ABS/CS, TR/CS, ET/CS, RC/CS in *U. lactuca* (Figure 3B,D,F,J, respectively).

**Figure 4:**
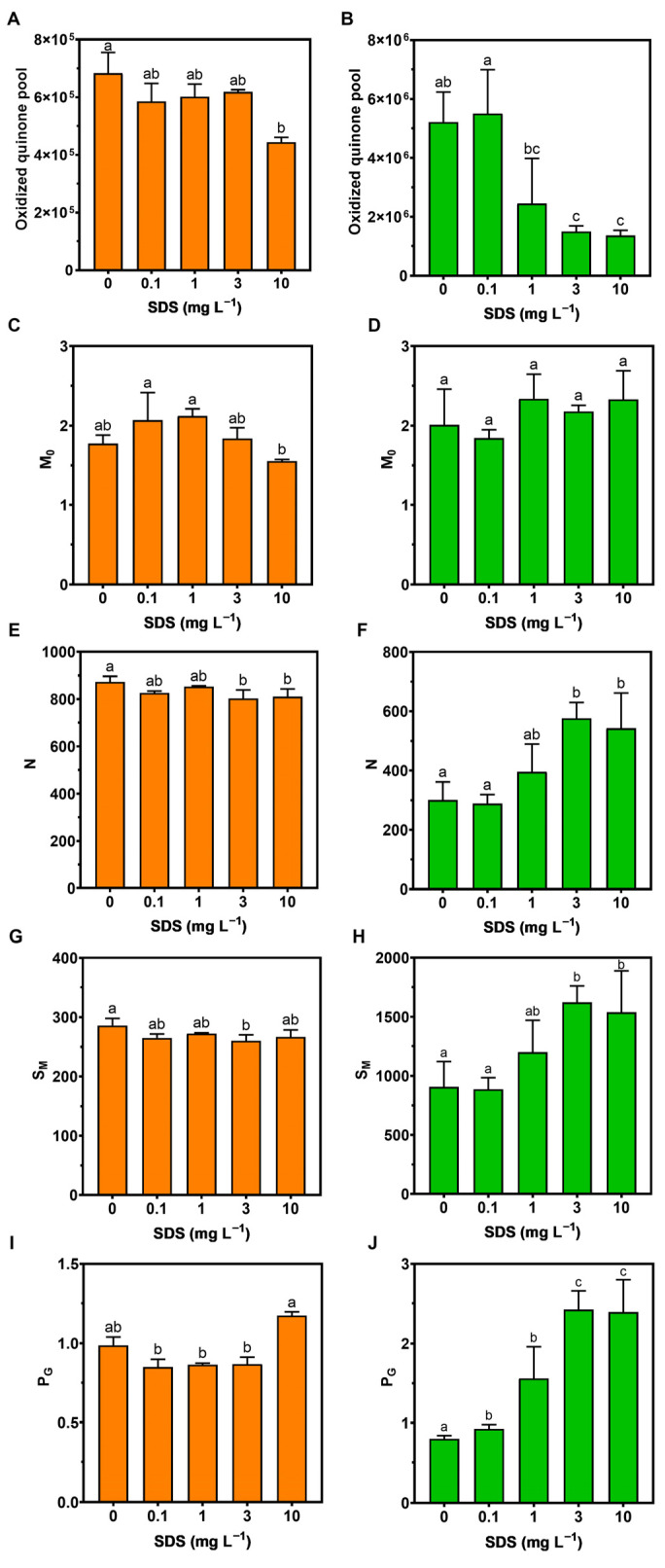
Parameters derived from OJIP transient curves in *Phaeodactylum tricornutum* (**A**,**C**,**E**,**G**,**I**) and *Ulva lactuca* (**B**,**D**,**F**,**H**,**J**) following 48-h of exposure to SDS increasing concentrations: (**A**,**B**) Oxidized quinone pool size; (**C**,**D**) Net rate of PS II RC closure (M_0_); (**E**,**F**) Turnover number of Q_A_ (N); (**G**,**H**) Corresponds to the energy needed to close all reaction centres (S_M_); (**I**,**J**) Grouping probability (P_G_) (mean ± s.d., *n* = 3 per treatment, different letters denote significant differences between treatments at *p* < 0.05).

Regarding the oxidized quinone pool, in *P. tricornutum,* a decrease occurred but was only significant at the highest SDS concentration (10 mg L^−1^) (Figure 4A), while in *U. lactuca* the decrease in this parameter was sensitive to SDS concentrations as low as 1 mg L^−1^ (Figure 4B). In the net rate of the PS II RC closure (M_0_), no significant differences were observed relative to the control in either species (Figure 4C,D). On the other hand, Quinone A turnover (N) presented significant differences with the control in the highest SDS tested concentrations (3 and 10 mg L^−1^) in both species, although with different patterns, decreasing in *P. tricornutum* (Figure 4E) and increasing in *U. lactuca* (Figure 4F).

Concerning the energy necessary to close all RCs (S_M_), *P. tricornutum* only showed a significant decrease at the SDS concentration of 3 mg L^−1^ (Figure 4G), while in *U. lactuca,* a similar pattern as the previous parameter was observed (Figure 4H). On the other hand, although the grouping probability between the two PS II units (P_G_) showed no significant changes in the samples subjected to SDS regarding the control in *P. tricornutum* (Figure 4I), this parameter showed a significant increase along the SDS concentration range in *U. lactuca* (Figure 4J).

**Figure 5:**
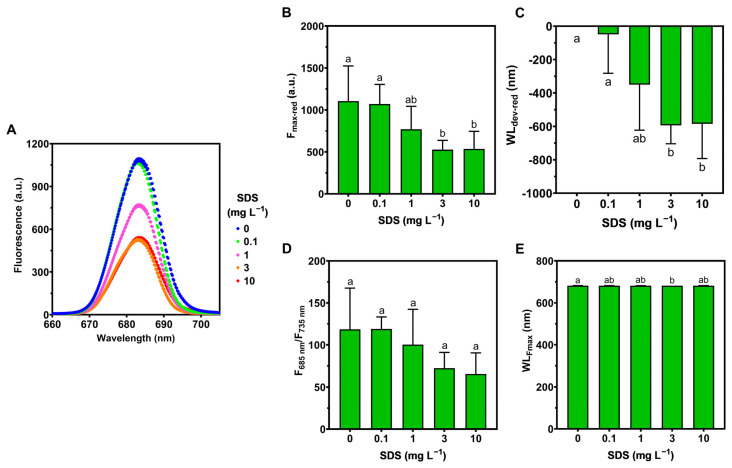
Red region laser-induced fluorescence in *Ulva lactuca* following a 48-h exposure to SDS increasing concentrations (**A**) (mean, *n* = 3) and measured parameters; (**B**) maximum fluorescence in the red region (F_max-red_); (**C**) wavelength deviation of the maximum fluorescence peak in the red region (WL_dev-red_); (**D**) red/far-red fluorescence ratio (F_685_/F_735_); (**E**) wavelength of the maximum fluorescence in the red region (WL_Fmax_) (mean ± s.d., *n* = 3 per treatment, different letters denote significant differences between treatments at *p* < 0.05).

The laser-induced fluorescence in the red region, measured only in *U. lactuca* (Figure 5A), showed significant decreases in the maximum fluorescence in the red region (F_max-red_; Figure 5B), and in the wavelength deviation of the maximum fluorescence peak (WL_dev-red_; Figure 5C) after exposure to SDS concentrations of 3 and 10 mg L^−1^. On the other hand, the red/far-red fluorescence ratio (F_685_/F_735_; Figure 5D), which varies inversely with chlorophyll content, showed no significant changes (although presenting a decreasing trend with increasing SDS concentration), and the wavelength of the maximum fluorescence in the red region (WL_Fmax_; Figure 5E) showed a significant decrease only when exposed to an SDS concentration of 3 mg L^−1^.

### 3.3. Pigment Composition

Pigment composition did not present significant changes in response to increasing SDS concentrations relative to the control in *P. tricornutum* (Table 2).

On the other hand, the changes observed in the pigment profile of *U. lactuca* showed significant decreases with exposure to SDS increasing concentrations in all measured pigments, except for antheraxanthin, which showed no significant changes (Table 3). Pigments with significant decreases showed changes at SDS concentrations equal to or higher than 1 mg L^−1^, although pheophytin *a* only decreased after exposure to SDS concentrations equal to or higher than 3 mg mL^−1^.

### 3.4. Fatty Acid Profile

**Figure 6:**
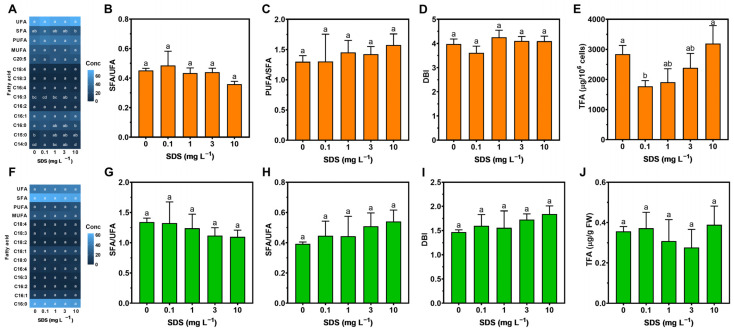
Fatty acids profiles (**A**,**F**); fatty acid ratios (saturated to unsaturated fatty acids ratio (SFA/UFA) (**B**,**G**); polyunsaturated to saturated fatty acids ratio (PUFA/SFA) (**C**,**H**); and double-bound index (DBI) (**D**,**I**)); and total fatty acids (TFA) (**E**,**J**) in *Phaeodactylum tricornutum* (top figures) and *Ulva lactuca* (bottom figures) after 48-h exposure to SDS increasing concentrations (mean ± s.d., *n* = 3 per treatment, different letters denote significant differences between treatments at *p* < 0.05).

SDS exposure triggered changes to the fatty acid profiles of *P. tricornutum*, namely increases in the concentrations of myristic acid (C14:0) after exposure to 0.1 and 3 mg L^−1^ SDS, and in the concentrations of triunsaturated hexadecatrienoic acid (C16:3n-4) after exposure to the highest SDS concentration (Figure 6A). On the other hand, palmitic acid (C16:0) was negatively impacted after exposure to the highest SDS concentration. Nevertheless, the SFA/UFA and PUFA/SFA ratios and the DBI showed no significant changes (Figure 6B–D) but a significant decrease in total fatty acids (TFA) was observed in the diatom after exposure to the three lowest SDS concentrations (Figure 6E).

In *U. lactuca*, no significant changes were detected in the fatty acid profiles upon SDS exposure (Figure 6F) nor in the evaluated ratios and TFA (Figure 6G–J).

### 3.5. Oxidative Stress

**Figure 7:**
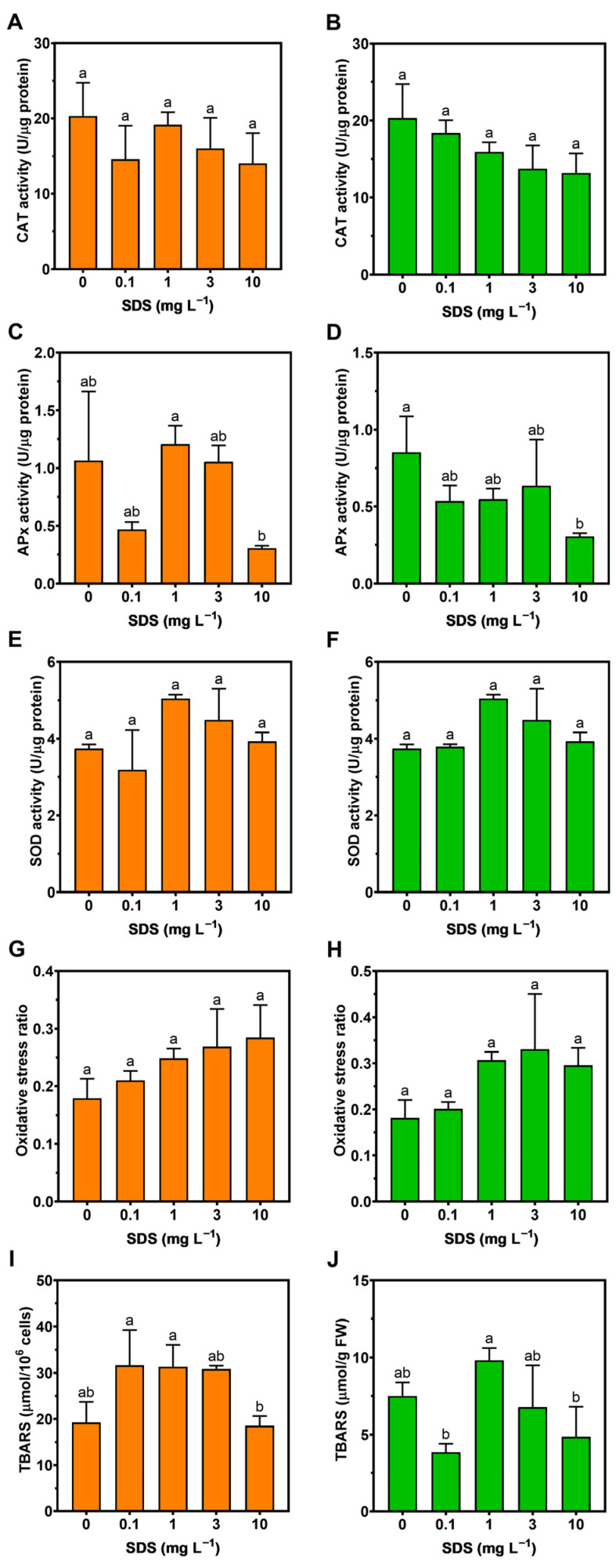
Catalase (CAT, (**A**,**B**)); ascorbate peroxidase (APx, (**C**,**D**)); and superoxide dismutase (SOD, (**E**,**F**)) enzymatic activities, oxidative stress ratio (**G**,**H**); and lipid peroxidation products (TBARS, (**I**,**J**)) in *Phaeodactylum tricornutum* (left figures) and *Ulva lactuca* (right figures) following a 48-h exposure to SDS rising concentrations (mean ± s.d., *n* = 3 per treatment, different letters denote significant differences between treatments at *p* < 0.05).

Regarding oxidative stress biomarkers, in general, no dose-response trends could be observed in either species (Figure 7). Catalase (Figure 7A,B) and SOD (Figure 7E,F) showed no significant alterations in response to external SDS exposure. Considering the tested antioxidant enzymes, APX showed no significant differences in *P. tricornutum* (Figure 7C); on the other hand, a significant decrease was observed in *U. lactuca* at the highest SDS concentration (Figure 7D). Nevertheless, the oxidative stress ratio showed no significant changes in either species (Figure 7G,H), as was the case of the lipid peroxidation products (TBARS) (Figure 7I,J).

### 3.6. Energy Balance

Concerning energy balance, the carbohydrate content in *P. tricornutum* decreased significantly after SDS exposure at the highest concentrations (3 and 10 mg mL^−1^; Supplemental Figure S3A), but we observed no variation in *U. lactuca* (Supplemental Figure S3B). Lipids and proteins showed no statistically significant variations with SDS concentrations for either species, as was the case of available energy (Ea), energy consumption rate (electron transport system at the mitochondrial level, ETS), and cellular energy allocation (CEA) (Supplemental Figure S3C–L).

### 3.7. Biomarker Multivariate Analysis

**Figure 8:**
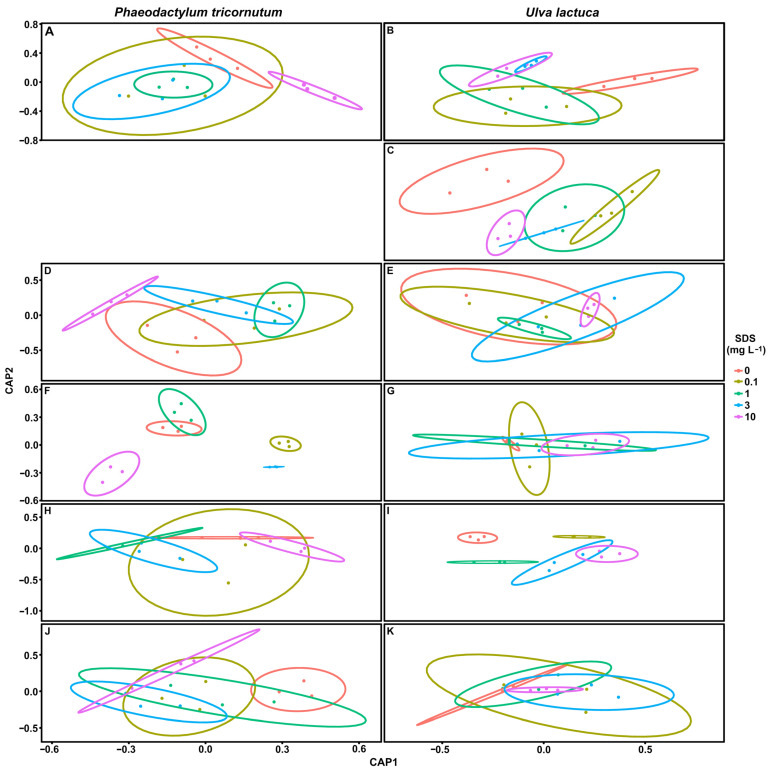
The plot of Canonical Analysis of Principal Coordinates (CAP) based on the photochemical traits (**A**,**B**); laser-induced fluorescence (**C**); pigment composition (**D**,**E**)’ fatty acid profile (**F**,**G**); oxidative stress (**H**,**I**) and energy balance (**J**,**K**) in *Phaeodactylum tricornutum* (left figures) and *Ulva lactuca* (right figures) following exposition to SDS over 48-h. The ellipses group samples with lower statistical distance are based on Euclidean resemblances.

The multivariate canonical classification was used to determine the accuracy of the measured parameters to identify different SDS concentration exposure effects in *P. tricornutum* and *U. lactuca* (Figure 8). Overall, in *P. tricornutum*, fatty acids data was more efficient (93.3%, Figure 8F) in distinguishing the applied concentrations in comparison with the photochemical traits (53.3%, Figure 8A), pigment composition (66.7%, Figure 8D), oxidative stress (53.3%, Figure 8H), or energy balance (53.3%, Figure 8J). In *U. lactuca*, oxidative stress data allowed the best separation between treatments (86.7%, Figure 8I), followed by photochemical traits (73.3%, Figure 8B) and LIF data (66.7%, Figure 8C), while pigment composition data (40.0%, Figure 8E), fatty acids profiles (33.3%, Figure 8G), and energy balance (33.3%, Figure 8K) did not allow a clear separation between SDS treatments. The overlap between ellipses and correspondent misclassification of these samples leads inevitably to a decrease in the overall canonical classification efficiency when those traits are used as biomarkers.

The photochemical and biochemical analysis of *P. tricornutum* and *U. lactuca* generated parameters with significant correlations in response to exposure to different SDS concentrations (Supplemental Figures S1 and S2, respectively). *Ulva lactuca* individuals exposed to SDS showed a higher percentage of biomarkers with significant correlations (45%) with the exogenous SDS dose applied when compared with ***P. tricornutum*** (42%) (Supplemental Table S1). The major difference among the species was detected at the biomarkers with enhanced values upon SDS exposure, with *U. lactuca* showing a higher relative abundance of these traits (19%) in comparison with the value observed for *P. tricornutum* (16%). Significantly, in *U. lactuca,* there is also a high number of correlated variables, especially within metabolic clusters (variables from the same type of analysis, e.g., pigments), when compared to this same pattern in *P. tricornutum*.

## 4. Discussion

The harmful effects of contaminants are dependent on their environmental concentration and on the sensitivity of the tested species [51,78], triggering multiple responses across species [79,80]. Moreover, there is an overlap between the SDS concentrations found in wastewater treatment plants, ranging between 1.15 mg L^−1^ and 10.6 mg L^−1^ [11,41] and the concentrations used in the present study, which shows biological effects in marine primary producers with possible toxic effects. Furthermore, alterations in the primary producers’ functioning and abundance may decrease the energy fluxes (e.g., carbohydrates, fatty acids) and oxygen production [26,27,28,81] and have a direct impact on marine communities. Hence, physiological, and biochemical changes in phototrophs can be used as biomarkers of pollutant exposure, such as changes in primary photochemistry [28], antioxidative responses [82,83], or fatty acid profiles [26,27].

### 4.1. Micro and Macroalgae Are Impacted by Environmentally Relevant SDS Concentrations

In this work, the model diatom *P. tricornutum* and the macroalgae *U. lactuca* showed differences in the several traits analysed to search for representative biomarkers, following exposure to increasing SDS concentrations during a 48-h period. It is known that the measurement of factors such as growth rate is a good indicator for the phytotoxicity determination of a contaminant in primary producers in this type of assay [84]. In response to the increasing exposure to SDS, a deceleration was observed in the growth rate of *P. tricornutum*, especially between the control and the highest applied concentration (10 mg L^−1^). Another work in marine diatoms (*Skeletonema costatum* and *P. tricornutum*) showed higher sensitivity to SDS exposure in comparison to freshwater green algae (*Pseudokirchneriella subcapitata*) at similar concentrations (0.3–7.9 mg L^−1^) [49]. Moreover, other studies reported that other green microalgae have a higher tolerance to SDS presenting a higher IC50, such as 30.2 mg L^−1^ (*Tetraselmis chuii*) [85] and 36.5 mg L^−1^ (*Raphidocelis subcapitata*) [86,87]. The higher sensitivity of *P. tricornutum* to SDS could be explained by diatom-extrinsic differences (e.g., cell culture parameters such as temperature, light intensity, water salinity, type of medium, SDS exposure duration) or diatom-intrinsic characteristics (e.g., lipid composition, SDS interaction with membrane lipid). In contrast, although *U. lactuca* first displayed a negative trend at 1 mg L^−1^, the growth rate was significantly stimulated at high SDS concentrations (3 and 10 mg L^−1^). Previous studies observed growth stimulation at surfactant concentrations usually greater than 2 mg L^−1^ [88]. In *Lemna minor*, concentrations of SDS up to 40 mg L^−1^ induced an increased growth rate, which could be the result of SDS serving either as a source of carbon, or, allowing a higher influx through moderate permeation effects [88,89]. Such growth can also occur in the environment since the presence of anionic surfactants in coastal areas led to an increase in algae productivity [90].

As stated, the concentration of anionic surfactants in aquatic environments has seasonal variation and is dependent on environmental conditions such as currents, the intensity of sea traffic, the frequency of diurnal sewage discharges, and the efficiency of wastewater treatment processes regarding these agents. Environmental concentrations of anionic surfactants have been reported to reach up to 10.6 mg L^−1^; of relevance being that their effects are dose-dependent [11,41]. Their detergent and solubilization ability are inherent to their capacity to form micelles when in solution. At low concentrations, they form monomers, in contrast with high concentrations, where the surfactant molecules aggregate to form clusters with the hydrophobic group located in the centre of the micelle and the hydrophilic group in contact with the solvent [91].

Exposure to a stress agent has energy costs, derived from the necessary metabolic effort required for the basic maintenance of the cell, leading to detrimental consequences regarding the growth and reproductive functions [92]. In addition, previous work showed that growth variations in *P. tricornutum* in response to a stressor are generally associated with impaired photosynthesis, and altered fatty acid content [27,28,47,48], often leading to reactive oxygen species (ROS) formation, which are the cellular response to stress signalling metabolites par excellence, responsible for opening and activating defence pathways. On the other hand, in *U. lactuca*, the negative effects of stress are not so visible, with the macroalgae showing tolerance to contamination and even the potential for remediation [40,93,94].

### 4.2. SDS Negatively Impacts Photochemistry and Pigment Content

Photochemical data showed an overall negative response to SDS exposure for both species. However, the reduction in the absorbed energy flux (ABS/CS) in *U. lactuca* in SDS concentration ranging between 1 and 10 mg L^−1^ led to a lower number of excitons generated; which in turn, led to lower numbers of electrons passing through the electron transport chain and the dissipation of energy (DI/CS), resulting in it remaining at its basal state. The decrease in ABS, TR and ET fluxes, which led to a decrease in dissipated energy, and the decrease in RC, may be counteractive measures to avoid PS II burnout and irreversible photoinhibition seen as the destruction of the D1 protein crucial to the repair cycle of PS II [95,96], which is in line with the observed decrease in pigments with increasing SDS exposure. The same variation was not observable in *P. tricornutum*, where no significant changes occurred in ABS/CS, which could lead to excess energy in the system since RC/CS and RC/ABS also decreased, with stressed cells not being able to cope with the same light intensities as healthy cells [97].

The donor site of the PS II, often known as the oxygen-evolving complex, is responsible for water-splitting, oxygen production and fuelling the quinone pool [98,99]. The size of the oxidized quinone pool significantly decreased in both species, although only in the highest SDS exposure for *P. tricornutum*; while in *U. lactuca*, it drastically decreased from 1 mg L^−1^. The obtained data shows that SDS exposure has a slight stress pressure on the quinone pool, which does not seem to be translated into a very significative biological negative effect in *P. tricornutum*, since only the QA turnover number (N) slightly decreased at the higher SDS concentrations. On the other hand, *U. lactuca* presents not only an increase in N at those SDS concentrations but also an increase in the relative pool size of plastoquinone (S_M_). These results could indicate that, in the case of exposure to higher SDS concentrations, the effects on the quinone pool could translate into enhanced formation of ROS [100]. However, the antioxidant system response presented minor variation in both phototrophs.

The grouping probability (P_G_) represents the connectivity between two PS II units and is inversely proportional to their overall health [57,97]. Although no significant changes occurred in *P. tricornutum*, an increase in P_G_ was observed in *U. lactuca* in a dose-response pattern, even at the lowest SDS concentration, which makes it a potential biomarker for SDS contamination. This pattern might be part of the mechanisms that *U. lactuca* uses in response to SDS since this increase would lead to a rise in ABS/CS and, since this was absent, it may be a strategy for maintaining the balance between the light and dark reactions, and the redox equilibrium between both photosystems [40,101]. This potential tolerance mechanism was further confirmed by the changes that occurred in the pigment content.

In *U. lactuca*, all pigments decreased, apart from antheraxanthin, which remained constant. Previously, several studies showed that, in *Ulva* spp., antheraxanthin accumulation is a common response to environmental stress, either by a contaminant [40], by desiccation [102], or by high light [103], serving as a protection of the thylakoid membrane. Since the growth rate was stimulated by increasing SDS exposure, pigment decrease may also be part of the tolerance mechanism, as *U. lactuca* was previously reported as a potential remediator [94]. On the other hand, pigment analysis revealed that *P. tricornutum* showed no significant changes when exposed to SDS. In this microalga, photosynthesis is often impaired or enhanced by variations in the pigments that participate in the fucoxanthin–chlorophyll proteins (FCPs), namely chlorophyll *a* and *c* and fucoxanthin, but also the pigments involved in photoprotection mechanisms such as β-carotene, diadinoxanthin and diatoxanthin [104,105]. Since no variation occurred in the pigment profiles, this may explain the poor photochemical performance, with excess radiation impairing photosynthesis.

### 4.3. Energy Balance and Fatty Acid Profiles

The stress exerted on photosynthesis can affect the pathways that use photosynthetic by-products. Regarding energy allocation and consumption, there were no significant changes in either species. Nevertheless, in *P. tricornutum*, a significant decrease was detected towards carbohydrate content, probably associated with constraints related to light-dependent reactions. In other diatom species, the depletion in stored carbohydrates can be associated with impairments regarding the thylakoid membrane integrity since the photosynthetic capability and the plasmalemma controls between the intra- and extracellular contents were compromised [48,106,107]. These alterations in photosynthesis efficiency could be related to the lipid environment surrounding the ETC [48]. Microalgae tend to vary their lipid content to overcome stress, though this response is species-specific [108]. Membrane lipids have distinctive signatures when it comes to fatty acid composition, and it is possible to infer changes in relative lipid abundance through variations in lipid-specific fatty acids. In *P. tricornutum*, the chloroplastidial fatty acid C16:3n-4 increased at the highest SDS concentration, and its presence is often associated with plastidial galactolipids, indicating a possible increase of these compounds that participate in FCP and ET stabilization and OEC dissociation prevention [106,109,110]. Another important lipid that constitutes thylakoid membranes is sulfoquinovosyldiacyl-glycerol, which is rich in C16:0 and C16:1 [48,110], the former showing a tendency to decrease. In SDS-treated diatoms, despite no significant variance, most individual FAs were observed; differences are highlighted when they are grouped together, resulting in an increase in UFA, being directly connected to membrane fluidity. This increase in the overall FA double bonds, together with the decrease observed in the percentage of the major saturated fatty acid C16:0, could be related to an inhibition of de novo FA synthesis and an enhancement of elongase and desaturase activities. Coupled with the results of the oxidative stress biomarkers, it is possible to conclude that mild stress on *P. tricornutum* was mostly felt at the chloroplast level but rapidly counteracted by changes in absorption and fatty acid unsaturation. On the other hand, the absence of changes in lipid composition in *U. lactuca* is in line with the abovementioned results, showing that SDS imposed almost no stress on *U. lactuca*, as previously observed [93].

### 4.4. Biomarker Potential in SDS Biomonitoring

Lastly, the application of CAP analyses was successful in identifying distinct levels of SDS-induced stress, mainly via lipid metabolism in *P. tricornutum* and photochemistry through LIF in *U. lactuca*. According to this approach, the fatty acid profile efficiently separated and classified four different concentration groups in *P. tricornutum*, providing a more efficient indicator of exposure than photochemical data, which was expected, due to SDS emulsifier properties [6]. On the other hand, bio-optical data allowed for a separation of *U. lactuca* into two groups (low and high SDS concentration), while oxidative stress, particularly APx activity, also allowed its separation into four groups. Variations in fatty acid profile and oxidative stress parameters in response to xenobiotics were observed previously [40] and present a potential use as biomarkers. This is important, since the same emergent pollutants can have different effects on different algae (e.g., growth inhibition, antioxidative activity) and, therefore, change the diversity of marine ecosystems and trophic chains’ nutritional value. In terms of classical biomarker assessment, a dose-effect relationship of individual biomarkers versus SDS dose applied; *U. lactuca* displayed a higher number of biophysical and biochemical traits with potential roles as biomarker candidates towards SDS exposure, belonging to quite different metabolic pathways and increasing, therefore, its usefulness for ecotoxicological applications and its use by different research groups focused on different evaluation techniques.

Overall, the diatom *P. tricornutum* and the macroalgae *U. lactuca* were exposed to environmentally relevant concentrations of SDS [11,41] and, to the best of our knowledge, this was the first extensive comparison of SDS effects on marine phototrophs’ metabolism aimed at developing biomarkers, encouraging further work to unravel not only other physiological alterations but also other genetic regulations that might occur in response to SDS toxicity that can be used as biomarkers.

## 5. Conclusions

The present work demonstrated how SDS concentrations found in natural aquatic environments affect multiple processes in marine autotrophs. Moreover, it showed the impacts this xenobiotic can have at the base of the food chain, and how SDS affects diatoms and macroalgae differently. The macroalgae *Ulva lactuca* presented many changes in photochemical biomarkers obtained by non-invasive, easy-to-use, high-throughput screening techniques. Nevertheless, *P. tricornutum* had a more classical dose-response to SDS, where changes to the fatty acid profile were the best biomarkers to identify the level of exposure on this microalga. These responses further support the use of this species as a suitable model for ecotoxicological assessments.

## Figures and Tables

**Table 1:** Fluorometric analysis parameters characterization.

OJIP-Test	
Area	Corresponds to the oxidized quinone pool size available for reduction and is a function of the area above the Kautsky plot
N	Reaction centre turnover rate
S_M_	Corresponds to the energy needed to close all reaction centres
M_0_	The net rate of PS II RC closure
P_G_	Grouping probability between the two PS II units
ABS/CS	Absorbed energy flux per cross-section
TR/CS	Trapped energy flux per cross-section
ET/CS	Electron transport energy flux per cross-section
DI/CS	Dissipated energy flux per cross-section
RC/CS	Number of available reaction centres per cross-section
RC/ABS	Reaction centre II density within the antenna chlorophyll bed of PS II

**Table 2:** *Phaeodactylum tricornutum* pigment profile following a 48-h exposure to SDS increasing concentrations. Pigments include chlorophyll *a* (Chl *a*), chlorophyll *c* (Chl *c*), pheophytin *a* (Pheo *a*), β-carotene (β-car), fucoxanthin (Fx), diadinoxanthin (Dx), and diatoxanthin (Dt) content. Average ± s. d., *n* = 3, different letters indicate significant differences between treatments at *p* < 0.05.

	SDS Concentration (mg L^−1^)				
Pigments (µg × 10^−8^ cell^−1^)	0	0.1	1	3	10
Chl *a*	39.32 ± 1.11 ac	50.83 ± 4.47 ab	56.74 ± 3.89 ab†	52.27 ± 2.15 ab	37.04 ± 3.10 c
Chl *c*	4.40 ± 1.44 a	5.15 ± 0.50 a†	4.71 ± 1.54 a	3.40 ± 1.59 a	1.49 ± 0.13 a
Pheo *a*	1.03 ± 0.33 ab	1.74 ± 0.87 a	2.05 ± 0.15 a†	1.12 ± 0.35 ab	0.17 ± 0.06 b
β-carot	3.57 ± 0.91 ab	4.92 ± 1.06 a	6.06 ± 0.71 a†	4.32 ± 0.56 ab	1.21 ± 0.02 b
Fx	9.73 ± 3.79 ab	8.95 ± 5.83 b	5.30 ± 1.37 b	14.49 ± 2.1 ab	22.20 ± 2.17 a†
DD	7.19 ± 1.18 a	10.03 ± 2.02 ab	11.48 ± 0.74 b†	8.93 ± 0.62 ab	7.63 ± 0.66 a
DT	6.96 ± 1.33 a	7.15 ± 2.79 a	5.66 ± 1.13 a	7.59 ± 0.98 a†	7.59 ± 1.58 a

† indicates the highest value.

**Table 3:** *Ulva lactuca* pigment profile following a 48-h exposure to SDS increasing concentrations. Pigments include chlorophyll *a* (Chl *a*), chlorophyll *b* (Chl *b*), pheophytin *a* (Pheo *a*), auroxanthin (Auro), Antheraxanthin (Anthera), β-carotene (β-carot), lutein (Lut), violaxanthin (Viola), and zeaxanthin (Zea) content. Average ± s.d., *n* = 3, different letters indicate significant differences between treatments at *p* < 0.05.

	SDS Concentration (mg L^−1^)				
Pigments (µg g^−1^ FW)	0	0.1	1	3	10
Chl *a*	66.03 ± 54.59 a†	55.05 ± 43.49 ab	18.63 ± 7.39 bc	10.87 ± 7.62 c	9.86 ± 0.45 c
Chl *b*	46.18 ± 37.69 a†	39.35 ± 31.28 ab	12.45 ± 4.58 bc	7.41 ± 5.26 c	6.30 ± 0.14 c
Pheo *a*	0.67 ± 0.92 a†	0.56 ± 0.63 a	0.09 ± 0.03 a	0.01 ± 0.02 b	0.00 ± 0.00 b
Auro	3.69 ± 2.60 a†	3.34 ± 2.26 a	1.14 ± 0.55 b	0.79 ± 0.54 b	0.79 ± 0.29 b
Anthera	3.82 × 10^−12^ ± 2.12 × 10^−12^ a	2.36 × 10^−12^ ± 1.06 × 10^−12^ a	1.00 × 10^−12^ ± 0.22 × 10^−12^ a	3.68 × 10^−12^ ± 3.77 × 10^−12^ a	4.60 × 10^−12^ ± 1.19 × 10^−12^ a†
β-carot	0.90 ± 0.42 a†	0.74 ± 0.48 a	0.31 ± 0.18 b	0.20 ± 0.12 b	0.23 ± 0.10 b
Lut	4.95 ± 3.75 a†	4.35 ± 3.65 ab	1.64 ± 0.49 bc	0.93 ± 0.57 c	1.00 ± 0.20 c
Viola	1.22 ± 0.89 a†	0.95 ± 0.60 ab	0.46 ± 0.30 bc	0.19 ± 0.17 c	0.13 ± 0.08 c
Zea	0.95 ± 0.45 a†	0.78 ± 0.51 a	0.33 ± 0.19 b	0.21 ± 0.13 b	0.24 ± 0.11 b

† indicates the highest value.

## Data Availability

Not applicable.

## Supplementary Materials

The following supporting information can be downloaded at: https://www.mdpi.com/article/10.3390/toxics10120780/s1, Figure S1: Heatmap of correlations in *Phaeodactylum tricornutum*; Figure S2: Heatmap of correlations in *Ulva lactuca*; Figure S3: Energy balance in *Phaeodactylum tricornutum* and in *Ulva lactuca*; Table S1: Percentage of biomarkers displaying SDS dose-effect significant correlations.
