# Supplementary material for: Ecotoxicological Effects of the Anionic Surfactant Sodium Dodecyl Sulfate (SDS) in Two Marine Primary Producers: Phaeodactylum tricornutum and Ulva lactuca
Source: Toxics. 2022 Dec 13;10(12):780. doi: 10.3390/toxics10120780 (PMC9785791; doi:10.3390/toxics10120780)
Supplement: Supplementary file 1 [file toxics-10-00780-s001.zip › toxics-2068469-supplementary.pdf]

Supplemental Figures

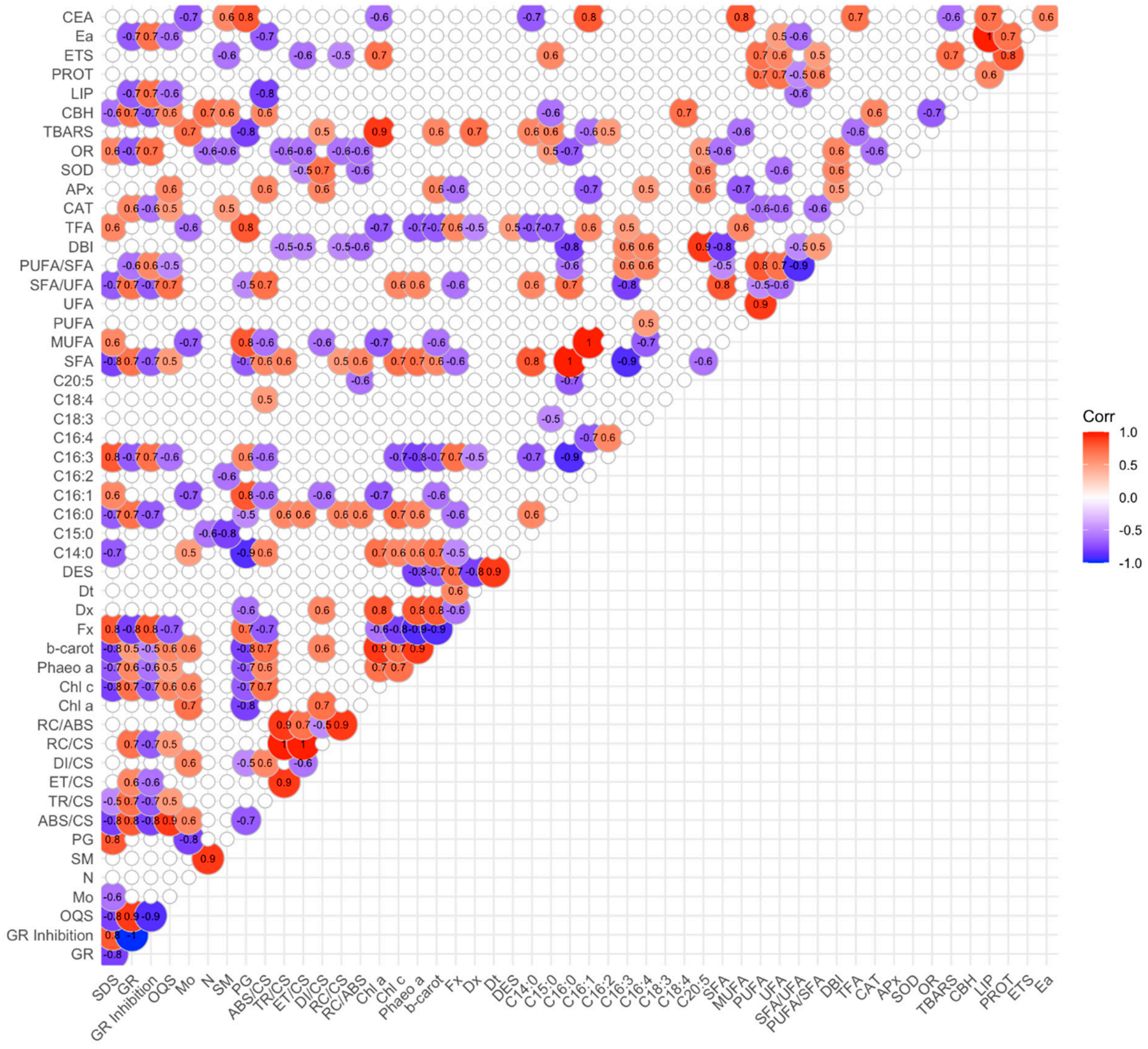

Figure S1. Heatmap of significant ($p < 0.05$) Spearman correlations of the measured parameters in *Phaeodactylum tricornutum* following 48-h exposure to increasing SDS concentrations.

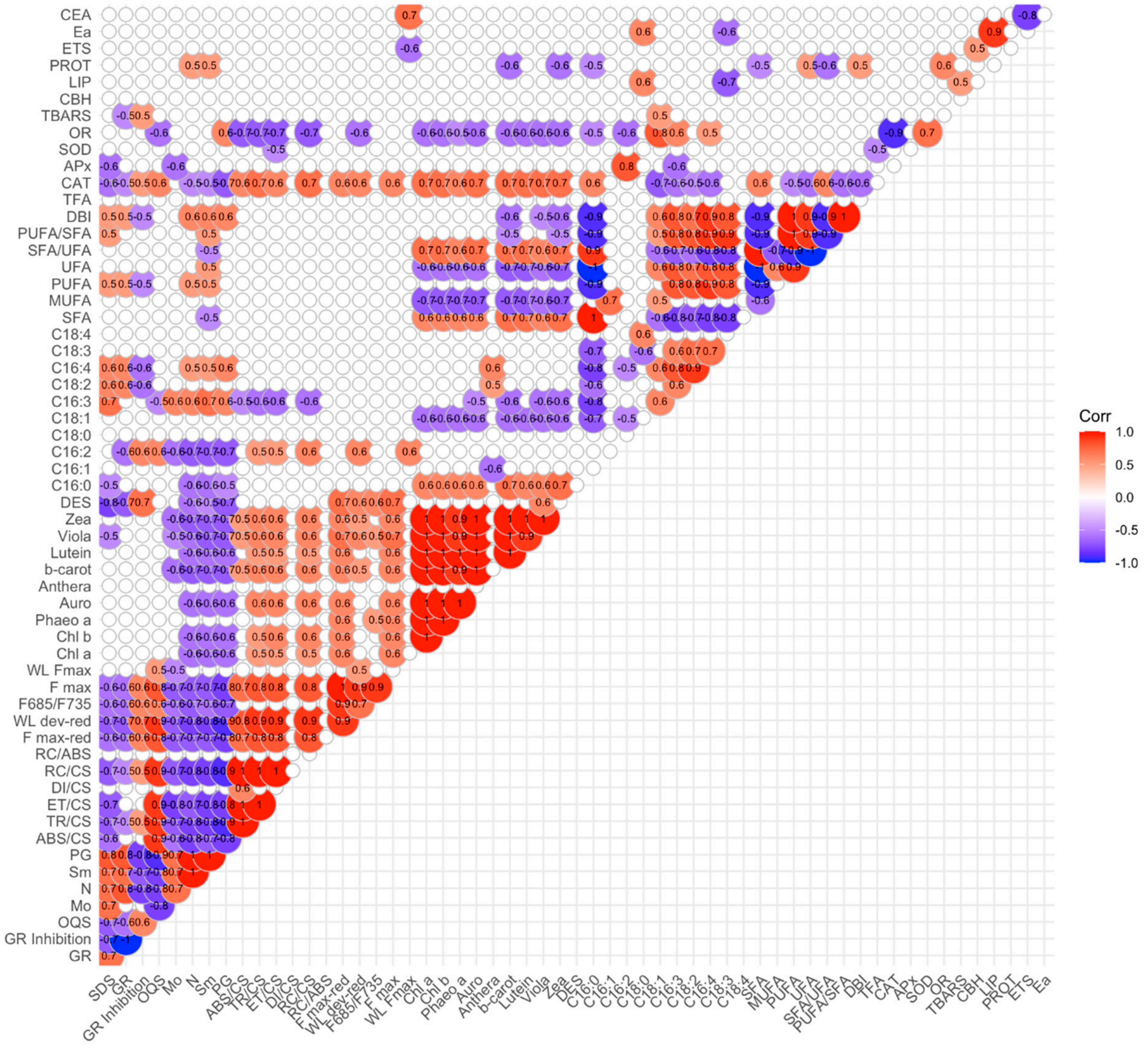

Figure S2. Heatmap of significant ($p < 0.05$) Spearman correlations of the measured parameters in *Ulva lactuca* following 48-h exposure to increasing SDS concentrations.

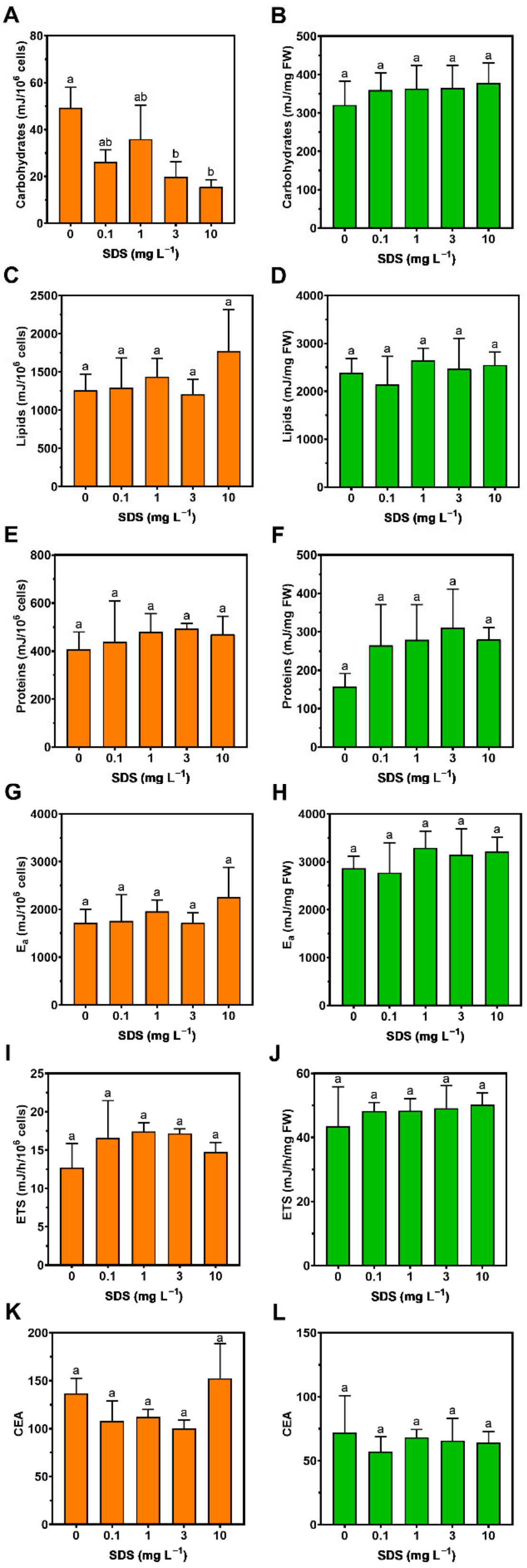

Figure S3. Carbohydrates (A,B), lipids (C,D), proteins (E,F), available energy (Ea; G,H), electron transport system at the mitochondrial level (ETS; I,J), and cellular energy allocation (CEA; K,L) changes in *Phaeodactylum tricornutum* (left figures) and in *Ulva lactuca* (right figures) after 48-h exposure to SDS (mean ± s.d., n = 3 per treatment, different letters denote significant differences between treatments at $p < 0.05$).

Supplemental Table

Table S1. Percentage of biomarkers displaying SDS dose-effect significant correlations, considering either enhanced or inhibited significant correlations in the two tested species.

	SDS Effect			
Species	Biomarkers with effect (%)	Enhanced (%)	Inhibited (%)	Total biomarkers assessed
Phaeodactylum tricornutum	42	16	26	50
Ulva lactuca	45	19	26	57
